# Cognitive impairment in comorbid MDD and OSA: the dual effects of intermittent hypoxia and sleep fragmentation

**DOI:** 10.3389/fpsyt.2026.1687180

**Published:** 2026-01-21

**Authors:** Jiajia Zhang, Shuangshuang Ma, Tianqin Xie, Mingming Zheng, Shuai Ding, Jiakuai Yu, Peng Zhu, Daomin Zhu

**Affiliations:** 1 Department of Sleep Medicine, Affiliated Psychological Hospital of Anhui Medical University, Hefei, China; 2 Department of Sleep Medicine, Anhui Mental Health Center, Hefei, China; 3 Department of Sleep Medicine, Hefei Fourth People's Hospital, Hefei, China; 4 School of Nursing, Anhui Medical University, Hefei, China; 5 Department of Maternal, Child and Adolescent Health, School of Public Health, Anhui Medical University, Hefei, China

**Keywords:** intermittent hypoxia, major depressive disorder (MDD), obstructive sleep apnea (OSA), P300, sleep continuity disruption

## Abstract

**Objectives:**

Major depressive disorder (MDD) and obstructive sleep apnea (OSA) exhibit an elevated comorbidity rate. It is posited that in MDD, comorbid OSA exacerbates cognitive impairment through mechanisms including intermittent hypoxia and sleep continuity disruption (sleep architecture disruption and sleep fragmentation).

**Methods:**

This cross-sectional study eventually recruited 245 patients aged 18 to 60 years. The cohort included patients with MDD (n=136), MDD with mild OSA (MDD-MO, n=75), and MDD with moderate-to-severe OSA (MDD-SO, n=34). Clinical symptoms, nocturnal sleep, and cognitive function were assessed using clinical psychological scales, polysomnography (PSG), and the event-related potential P300, respectively. We evaluated the intergroup differences in P300 components and their correlation with sleep parameters.

**Results:**

Compared to the other two groups, the MDD-SO group exhibited significant increases in the Oxygen Desaturation Index (ODI), the proportion of N1 sleep and Microarousal Index (p < 0.05). The MDD-SO group showed a marked reduction in N3 and REM sleep compared to both the MDD-MO and MDD groups (P < 0.05 for both). Additionally, P300 latency was significantly prolonged in the MDD-MO and MDD-SO groups relative to the MDD group (P < 0.001). Multiple linear regression identified AHI and ODI as a significant positive predictor of P2, N2, P3a and P3b latency, and Microarousal Index as a significant positive predictor of N1, P2, P3a, and P3b latency in MDD patients with OSA (all p < 0.05).

**Conclusion:**

The observed associations between prolonged P300 latency and elevated ODI/Microarousal Index raises the possibility that intermittent hypoxia and sleep fragmentation are underlying contributing factors. These two nocturnal disturbances may interact to worsen cognitive dysfunction in patients with MDD-OSA comorbidity.

## 1 Introduction

As a substantial worldwide health burden, major depressive disorder (MDD) affects a vast population across the globe (1, 2). Beyond the emotional symptoms, cognitive impairment is increasingly recognized as a central characteristic of MDD (3, 4), encompassing domains such as attention, memory, and executive function (5). It is noteworthy that cognitive impairment often persists even after the improvement or remission of emotional symptoms (6). This persistent impairment is closely associated with disease recurrence and poor long-term prognosis (7, 8). Obstructive sleep apnea (OSA) is a common chronic sleep-related respiratory disorder (9). A 2016 systematic review encompassing 12 studies reported that its prevalence in patients with depressive disorders was 36.3% in clinical settings and 19.8% in community cohorts (10). A more recent study (11) reaffirms this heightened comorbidity, estimating prevalence between 15% and 40% in this patient group—rates substantially higher than in the general population (11). Meanwhile, depression is highly prevalent among individuals with OSA. A meta-analysis of 34 studies (12) estimated a pooled prevalence of depressive symptoms of 35% in this population (12), substantially exceeding the reported rate of 4% in the general population (13). Collectively, these findings indicate a significant comorbidity between depression and OSA in clinical populations.

Research (14) has established that OSA adversely affects multiple cognitive domains (14), including memory, executive function, processing speed, and attention (15). Comorbid OSA can exacerbate the cognitive impairments in patients with MDD (16). For instance, a neuroimaging study (17) revealed that the comorbidity of depression and OSA is associated with more extensive alterations in brain structure (17). Jingjing Miao et al. (18) reported that comorbid OSA aggravates prospective memory impairment in depression (18). However, given the limitations of existing evidence-including small sample sizes, the inability of cross-sectional designs to establish causality, and age-restricted study populations-there is currently insufficient reliable evidence to confirm that OSA exacerbates the degree of cognitive impairment in patients with MDD. No consensus exists regarding the precise mechanisms underlying this impairment.

The classic pathophysiological model posits that cognitive impairment in patients with OSA is primarily driven by chronic nocturnal hypoxemia and sleep fragmentation (9). Literature (19) suggests that hypoxia, a key feature of OSA, can lead to structural brain changes. These neurological alterations underlie the patients’ cognitive deficits (19). Animal research (20, 21) has demonstrated that chronic intermittent hypoxia exposure leads to marked cognitive deficits in mice, accompanied by neuronal damage and apoptosis (20, 21). Furthermore, sleep disruption compromises critical overnight repair processes in the brain, thereby contributing to cognitive deficits (22). Employing a paradigm of intermittent tactile stimulation, researchers (23) induced chronic sleep fragmentation (SF) in mice. Subsequent behavioral testing revealed that SF-impaired mice displayed marked impairments in spatial learning and memory tasks (23). Emerging research (24) highlights that specific disturbances in sleep macro-architecture-such as reductions in deep sleep (N3) and REM sleep-are critically involved in the cognitive impairment observed in OSA (24). Similarly, Jia Wei et al. (25) have identified abnormal sleep architecture-characterized by reduced N3 sleep, increased N1 sleep, and prolonged wake after sleep onset (WASO) - as a potential marker of cognitive decline (25). Therefore, we propose that in patients with MDD, OSA may constitute an independent pathway that exacerbates cognitive impairment, operating through the mechanisms of intermittent hypoxia and sleep continuity disruption (sleep architecture disruption and sleep fragmentation).

The P300, an extensively studied event-related potential component, is a well-established neurophysiological index that reflects the neural efficiency of fundamental cognitive processes such as attention allocation, context updating, and stimulus evaluation (26). Owing to its non-invasiveness, cost-effectiveness, ease of operation, and high temporal resolution, P300 is a valuable neuroelectrophysiological tool (27, 28). To date, the P300 has been separately examined in individuals diagnosed solely with MDD and in patients with OSA. Research (29) consistently demonstrates that patients with depression exhibit prolonged P300 latency and reduced amplitude (29). In contrast, P300 abnormalities in OSA patients remain contentious. The literature reports (30) conflicting results regarding P300 characteristics in patients with OSA (30). Additionally, a meta-analysis by Jiang et al. (31) demonstrated that P300 latency exhibits greater stability and sensitivity than P300 amplitude in cognitive assessment (31).

This study investigates the relationships between intermittent hypoxia, disrupted sleep continuity, and cognitive deficits (indexed by P300) in patients with comorbid MDD and OSA.

## 2 Method

### 2.1 Participants and methodology

The study participants comprised patients diagnosed with major depressive disorder (MDD) who were consecutively recruited from the Sleep Disorders Department of the Fourth People’s Hospital of Hefei from January 2020 to June 2023. The diagnosis was established by two senior psychiatrists in line with the International Classification of Diseases (ICD-10) diagnostic criteria for MDD. To ensure diagnostic accuracy, all participants underwent further screening with the Mini-International Neuropsychiatric Interview (MINI). Study eligibility was restricted to individuals aged 18 to 60 years who demonstrated the ability to cooperate with all procedures-including polysomnography (PSG), clinical scale assessments, and P300 evaluations-and who provided written informed consent. Participants were excluded if they met any of the following conditions: a) mental disorders due to organic brain conditions or general medical diseases; b) intellectual disability or comorbid psychiatric disorders; c) a history of treatment for OSA; d) the presence of another sleep disorder, for example, central sleep apnea or narcolepsy; or e) long-term use of sedative-hypnotics or a history of substance dependence. A total of 307 individuals were initially approached. Of these, 39 were excluded for not meeting the inclusion criteria (specifically, 21 inpatients with neurological diseases, cardiovascular diseases, respiratory diseases; 7 inpatients with comorbid othermental disorders, such as schizophrenia; 4 inpatients suffered from other sleep disorders, such as narcolepsy; 5 inpatients had been using sedative-hypnotic drugs for a long time; 2 inpatients had received CPAP treatment before), and 23 inpatients were missing PSG or P300 data. Ultimately, 245 inpatients with depression were included in the final analysis. The flowchart of the participants is shown in **Supplementary Figure S1**. Trained nursing staff obtained anthropometric measurements (height and weight) to determine Body Mass Index (BMI, kg/m²). All participants underwent a comprehensive psychological assessment, which included the 17-item Hamilton Depression Rating Scale (HAMD-17), the Hamilton Anxiety Rating Scale (HAMA), and the Epworth Sleepiness Scale (ESS). Additionally, they completed ERP-P300 and polysomnography (PSG) evaluations. All assessments were conducted within the first week of hospitalization.

Based on polysomnography (PSG) results, patients were categorized into three groups: 1) the MDD group (AHI < 5 events/h, n = 136); 2) the MDD with mild OSA (MDD-MO) group (5 ≤ AHI < 15 events/h, n = 75); and 3) the MDD with moderate-to-severe OSA (MDD-SO) group (AHI ≥ 15 events/h, n = 34). Among them, the MDD subgroup consisted of 136 patients (40 men and 96 women), aged 38.2 ± 13.3 years; the MDD-MO subgroup consisted of 75 patients (23 men and 52 women), aged 48.4 ± 12.0 years; The MDD-SO subgroup consisted of 34 patients (16 women and 18 men) who had a mean age of 52.4 ± 9.5 years. All patients were treated with conventional antidepressant medications. Written informed consent was obtained from all participants following a detailed explanation of the study procedures. Ethical approval was acquired from the Ethics Committee of the Fourth People’s Hospital of Hefei prior to study initiation (Approval No: 2024-099-001; Date: March 12, 2024).

### 2.2 Clinical assessments

#### 2.2.1 17-item hamilton depression scale

The HAMD-17 is a well-validated, clinician-administered instrument that serves as a gold standard for quantifying depression severity (32). The HAMD-17 provides a total score spanning a spectrum of 0 to 54. Individual items are rated employing either three-or five-point Likert scales. A higher total score indicates more severe depression. According to conventional guidelines, a total score exceeding 24 is indicative of severe depressive symptoms (33).

#### 2.2.2 Hamilton anxiety rating scale

The HAMA is a clinician-rated instrument comprising 14 items designed to appraise the severity of anxiety, encompassing both somatic and psychic symptoms. The severity of each symptom is assessed employing a five-point Likert-type scale (0 to 4). A total score above 7, derived from the sum of all items, suggests clinically significant anxiety symptoms (34).

### 2.3 Sleep characteristics

#### 2.3.1 Polysomnography examination

Patients underwent two consecutive nights of video-polysomnography (vPSG) (Philips Respironics, Murrysville, PA, USA) to assess their nocturnal sleep. The initial night of monitoring was designated as the adaptation period. Subsequently, standard surface electrodes were applied to acquire electroencephalographic (EEG), electrooculographic (EOG), electromyographic (EMG), and electrocardiographic (ECG) data. Airflow was assessed via a nasal-oral thermistor, and thoracic-abdominal movements were recorded by plethysmographic belts. Finger pulse oximetry was used to assess blood oxygen saturation (SpO_2_), with polysomnography data being visually analyzed in compliance with the most recent American Academy of Sleep Medicine (AASM) criteria (35). The data obtained from the second polysomnographic monitoring session were analyzed employing established scoring criteria. In this study, we focused on the following key metrics: 1) sleep efficiency (SE; total sleep time divided by time in bed); 2) Sleep structure indicators: the relative duration (%) of N1, N2, N3, and REM sleep stages, and the proportion of the record scored as wakefulness; 3) Fragmentation index of sleep: Microarousal Index (The average number of micro-arousal events per hour of sleep); 4) Apnea-Hypopnea Index (AHI): Defined as the total number of apneas (complete cessation of airflow≥10 seconds) and hypopneas (≥30% reduction in airflow associated with ≥3% oxygen desaturation or an arousal) per hour of sleep (35), OSA severity was categorized as: mild (AHI≥5 and <15), moderate (AHI≥15 and <30), and severe (AHI≥30); 5)The oxygen desaturation index (ODI), defined as the number of oxygen saturation drops ≥3% per hour of sleep, was used to quantify the severity of nocturnal hypoxemia associated with OSA.

#### 2.3.2 Epworth sleepiness scale

The Epworth Sleepiness Scale (ESS) provides a subjective measure of daytime sleepiness by having individuals rate their dozing probability in eight specific situations. A total ESS score ≥10, on a scale from 0 to 24, is the established clinical threshold for excessive daytime sleepiness (36).

### 2.4 Recording of the P300 event-related potential

An auditory odd-ball paradigm was employed to elicit the P300 response in all participants. The study focused on the N1, P2, N2, and P3 (including subcomponents P3a and P3b) components evoked by the target stimuli, which served as the primary outcome measures. The Nicolet VikingQuest evoked potential/EMG system (USA) was employed for this study. Following the International 10-20 System, the recording electrode was placed at the Cz site, with reference electrodes affixed to both earlobes, Electrode-skin impedance was maintained below 5 kΩ. The target stimulus (T) was a 2000 Hz tone, presented with a probability of 20%, Participants were tasked with identifying and mentally counting the total number of these target tones; The non-target stimulus (NT) was a 1000 Hz tone, presented with a probability of 80%, Participants were asked to refrain from responding to these stimuli. The acoustic stimuli were delivered at a rate of 1/s, with a duration of 10 ms, The recording sensitivity was set at 5 μV. The analog bandpass filter was configured with a low-frequency cutoff at 750 Hz and a high-frequency cutoff at 2000 Hz. Between 100 and 150 sweeps were averaged for each recording.

Data collection for all participants was conducted in the event-related potential (ERP-P300) laboratory of the Sleep Disorders Department, Sessions were scheduled between 9:00 AM and 12:00 PM to minimize potential influences of circadian rhythms and fatigue on attention levels and cognitive performance. The laboratory is a spacious, quiet, and well-controlled environment with appropriate lighting, stable temperature, and proper electrical grounding, These conditions are maintained to minimize signal contamination and external interference during EEG recordings. Prior to the formal experiment, participants were fully briefed on the detailed procedures, A practice session was then administered to ensure task comprehension. The formal test commenced only after participants confirmed a clear understanding of all requirements. Throughout the recording, participants were instructed to minimize excessive body movements and eye blinks to ensure optimal signal quality. No other cognitively demanding tasks were administered immediately before this experiment.

**Figure 1:**
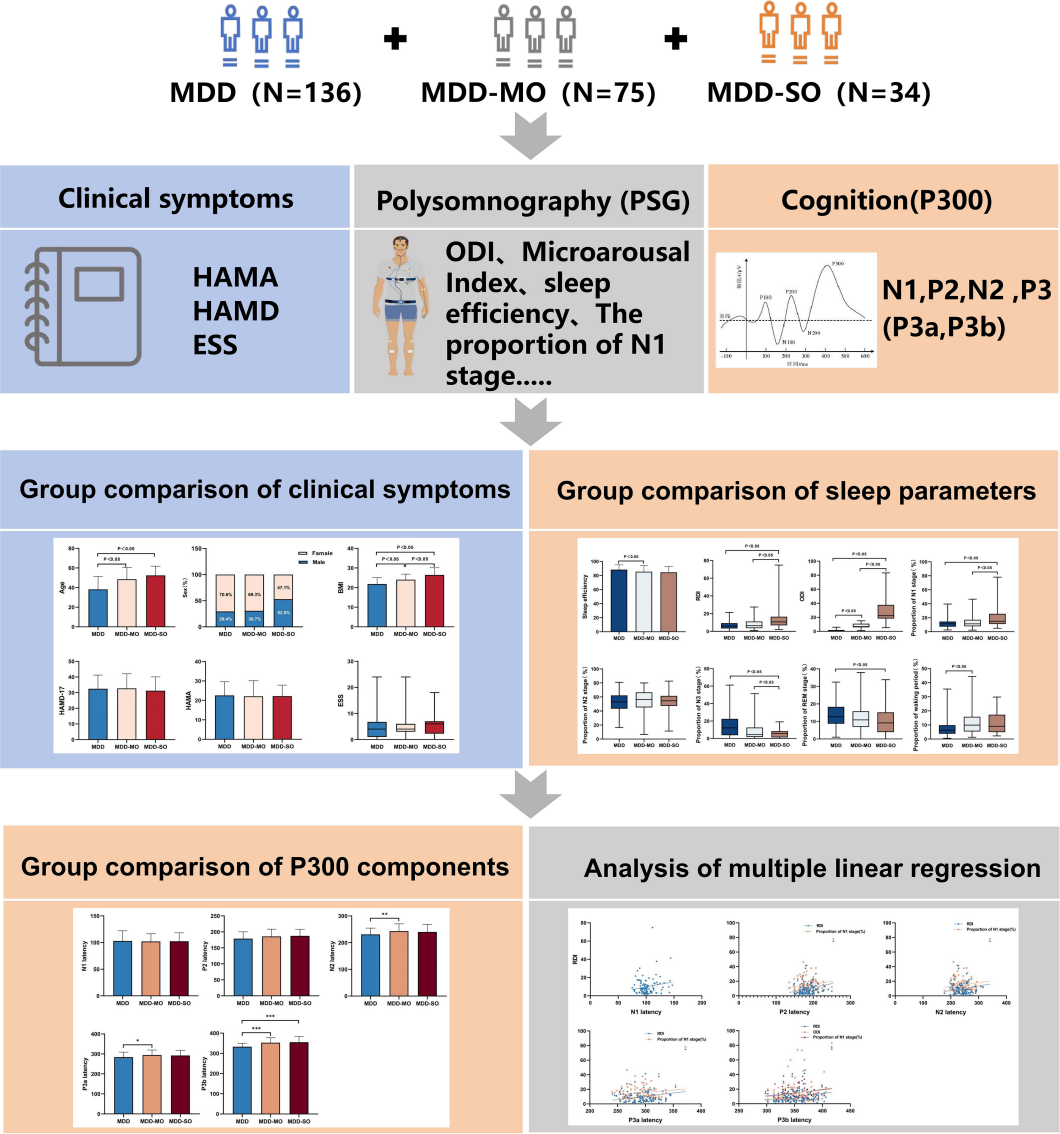
Research design and analytical procedure.We collected clinical symptoms, PSG and P300 data from 136 MDD patients, 75 MDD-MO and 34 MDD-SO patients.We used the Kruskal-Wallis test for inter-group comparisons and multiple linear regression to explore the correlations between hypoxia, sleep fragmentation and the P300 component. HAMA, Hamilton Anxiety Scale; HAMD, Hamilton depression scale; ESS, Epworth Sleepiness Scale; ODI, Oxygen desaturation Index; MDD, major depressive disorder; MDD-MO, MDD with mild OSA; MDD-SO, MDD with moderate to severe OSA.

EEG recordings were digitized at 1000 Hz and then preprocessed utilizing the EEGLAB environment (v14.1.0b) in MATLAB R2015b. The data were submitted to band-pass filtering (0.05-30 Hz), average re-referencing, and ocular artifact removal by means of Independent Component Analysis (ICA). The continuous EEG data were divided into 800-ms epochs, which included a 200-ms baseline period before stimulus onset (37). The P2 amplitude was quantified as the local positive maximum within the 120-300 ms epoch, time-locked to stimulus onset. Similarly, The N2 amplitude was quantified as the peak negativity in the 150-350 ms post-stimulus interval, and the P3 amplitude was operationalized as the peak positivity within the 260-500 ms window. P300 latency was measured as the duration from stimulus onset to the component’s peak amplitude (in milliseconds). The overall research design and analytical pipeline are summarized in **Figure 1**.

### 2.5 Statistical analyses

The statistical package SPSS (version 23.0) was utilized for data analysis. Continuous variables with normal distributions (e.g., demographic, clinical, PSG, P300 and ERP data) are summarized using mean and standard deviation (mean ± SD), One-way analysis of variance (ANOVA) was employed to compare these continuous variables between groups. Non-normally distributed continuous variables are summarized as median (IQR) and compared with the Kruskal–Wallis test, while categorical variables are presented as numbers and percentages. Data quantification was all accomplished using GraphPad Prism (8.0.0). Furthermore, a multiple linear regression was employed for examining the associations between PSG measures and P300 parameters, To eliminate the influence of known confounding factors, in the construction of the multiple linear regression model, we included age, gender, BMI, education level, smoking, drinking and pre-admission antidepressant use as covariates along with the core independent variable. All variables were entered simultaneously into the regression models, Multicollinearity among these predictors was formally assessed using the variance inflation factor (VIF), with a common threshold of VIF < 5 applied; Homoscedasticity was assessed by visually examining a scatter plot of standardized residuals against predicted values and by formally conducting the Breusch-Pagan test, The residual plot revealed no discernible pattern (e.g., funnel shape), and the Breusch-Pagan test was not significant (p > 0.05), supporting the assumption of homoscedasticity. A Bonferroni correction was applied to control the family-wise error rate and reduce Type I errors.The initial significance threshold was set to p=0.05 and was subsequently Bonferroni-corrected for multiple comparisons.

## 3 Results

### 3.1 Demographic and clinical characteristics

**Figure 2:**
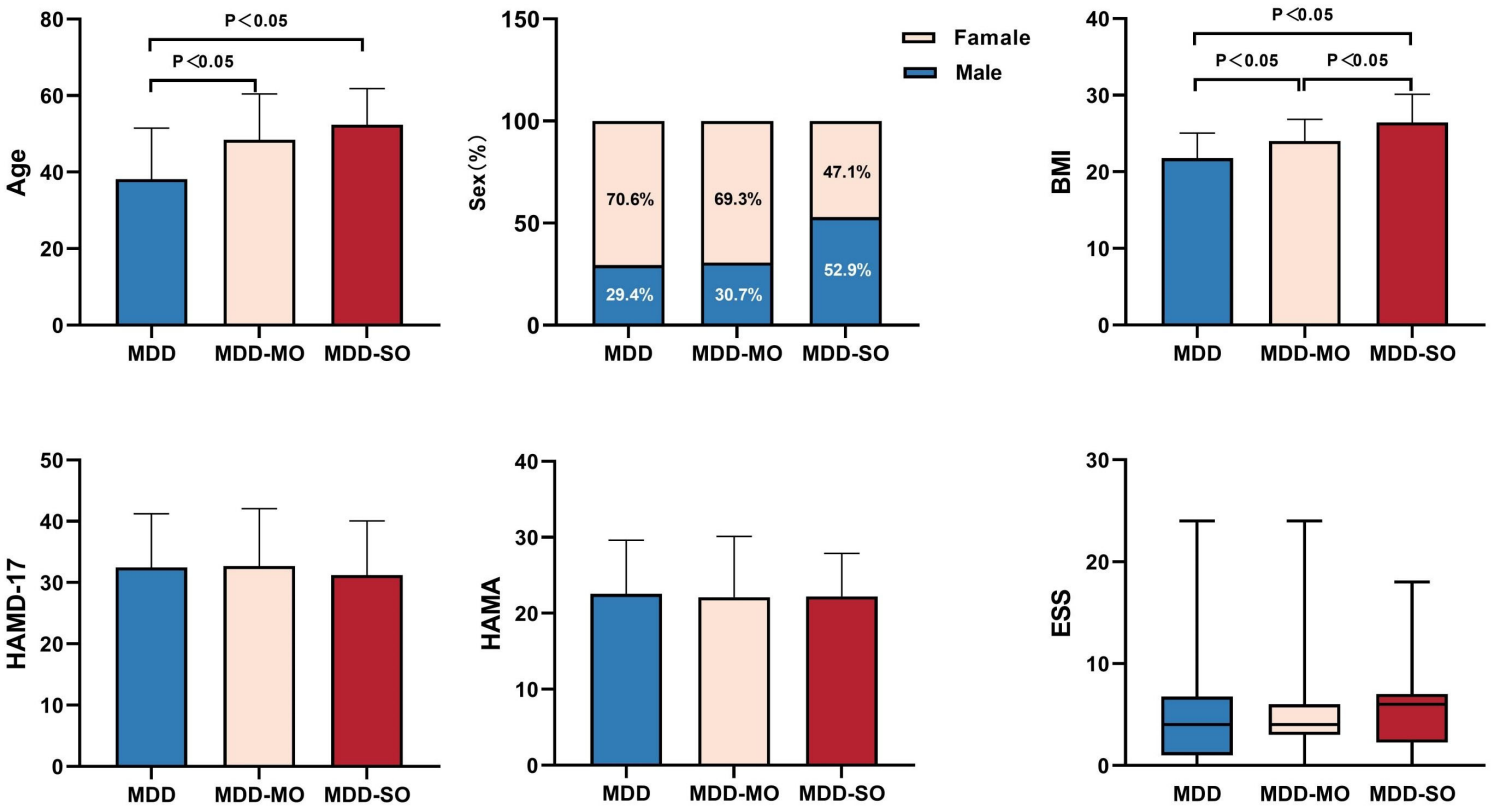
Comparison of demographic and clinical characteristics between groups. BMI, Body mass index; HAMD-17, Hamilton depression scale-17; HAMA, Hamilton Anxiety Scale; ESS, Epworth Sleepiness Scale.

Among the 245 patients recruited for the study on major depression, the cohort was divided into the following groups: MDD (n = 136), MDD-MO (n = 75), and MDD-SO (n = 34). Intergroup comparisons demonstrated significant differences in gender (p = 0.029), as well as in age and body mass index (BMI) (both p < 0.05). *Post-hoc* tests indicated that the MDD-MO and MDD-SO groups were significantly older than the MDD group (both p < 0.05). In addition, the MDD-MO and MDD-SO groups had significantly higher BMI indexes than the MDD group (p < 0.05) (**Figure 2**).

### 3.2 The differences of polysomnography characteristics in three groups

**Figure 3:**
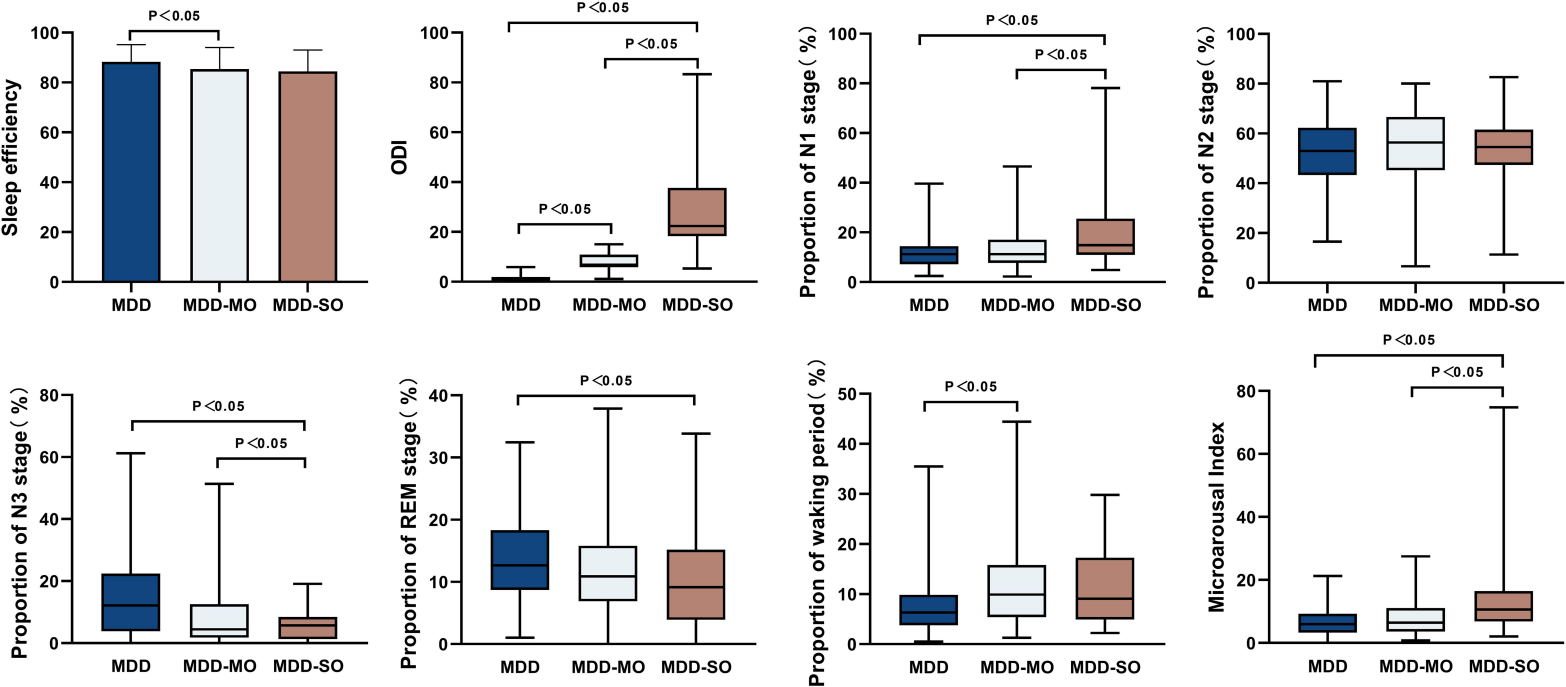
Inter-group comparison of polysomnography (PSG) features. ODI, Oxygen desaturation Index.

Sleep efficiency was significantly reduced in the MDD-MO group compared to the MDD group (85.4% vs. 88.3%, p < 0.05). The MDD-SO group had a significantly higher proportion of N1 stage sleep than both the MDD-MO and MDD groups (14.9% vs. 11.2% and 11.2%, respectively; p < 0.05). The proportion of N3 stage sleep was significantly lower in the MDD-SO group than in the MDD-MO and MDD groups (5.7% vs. 4.4% vs. 12.2%, p < 0.05). The MDD-SO group had a significantly lower proportion of REM sleep than the MDD group (9.1% vs. 12.7%, p < 0.05). The proportion of the waking period in the MDD group was significantly lower than that in the MDD-MO group (p < 0.05). Microarousal Index in the MDD-SO group were significantly higher than those in the MDD-MO and MDD groups (10.6 vs. 6.4 vs. 6.0, p <0.05). The AHI and ODI in the MDD-SO group were significantly higher than those in the MDD-MO and MDD groups (AHI: 23.7 vs. 7.5 vs. 1.0; ODI: 22.4 vs. 6.8 vs. 0.9; p <0.05). (**Supplementary Table S1**, **Figure 3**).

### 3.3 Differences in P300 component metrics among the three groups

**Figure 4:**
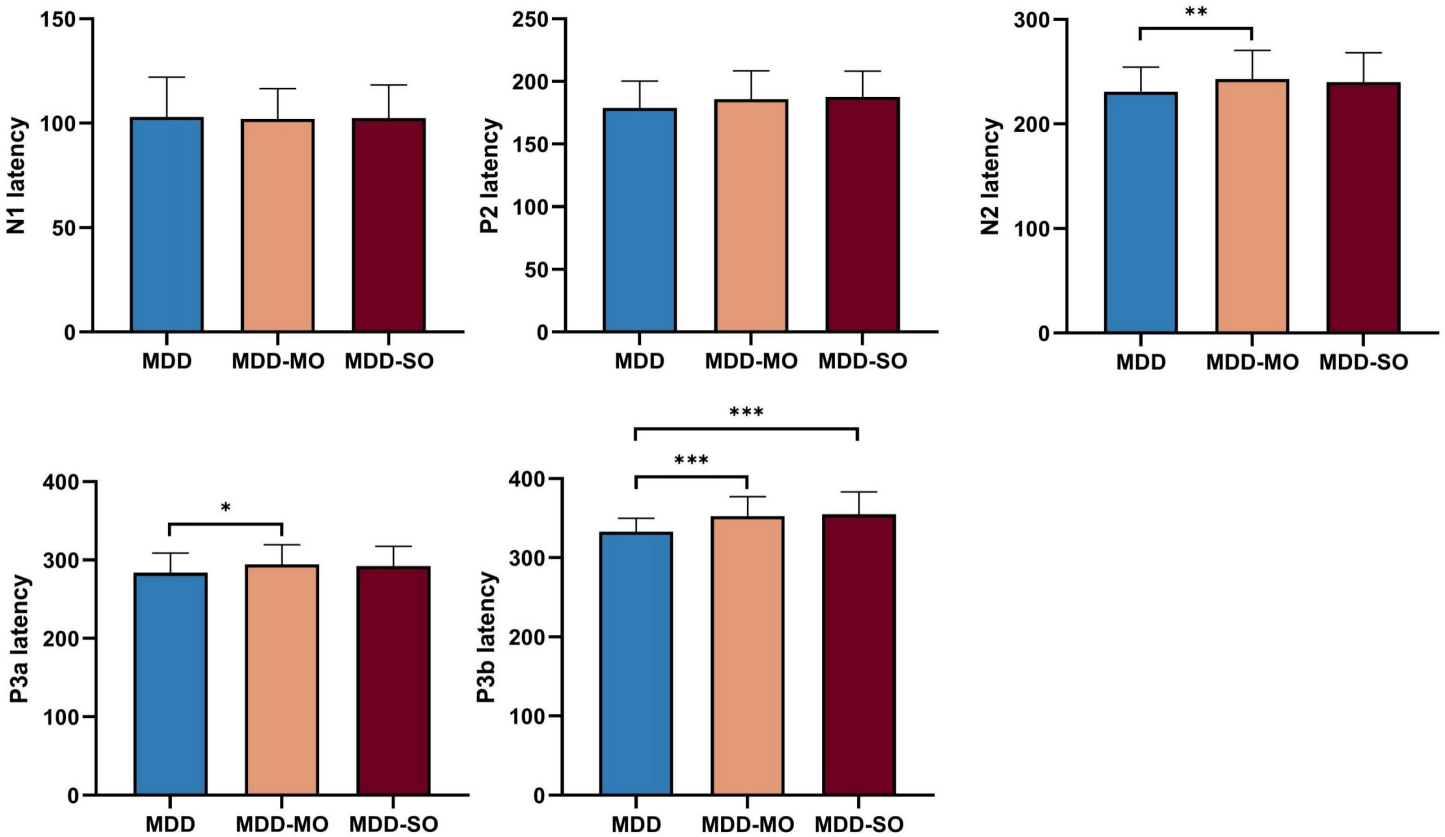
Inter-group comparison of P300 components. *P<0.05, **P<0.01, ***P<0.001.

Significantly longer latencies were observed in the MDD-MO group for N2 (p = 0.003) and P3a (p = 0.01) compared to the MDD group, respectively. While P3b latency was prolonged in both MDD-MO and MDD-SO groups (vs. MDD, p = 0.000), the N1 and P2 components showed no significant intergroup differences (p ;> 0.05) (**Figure 4**).

### 3.4 The association between P300 components and PSG metrics

**Figure 5:**
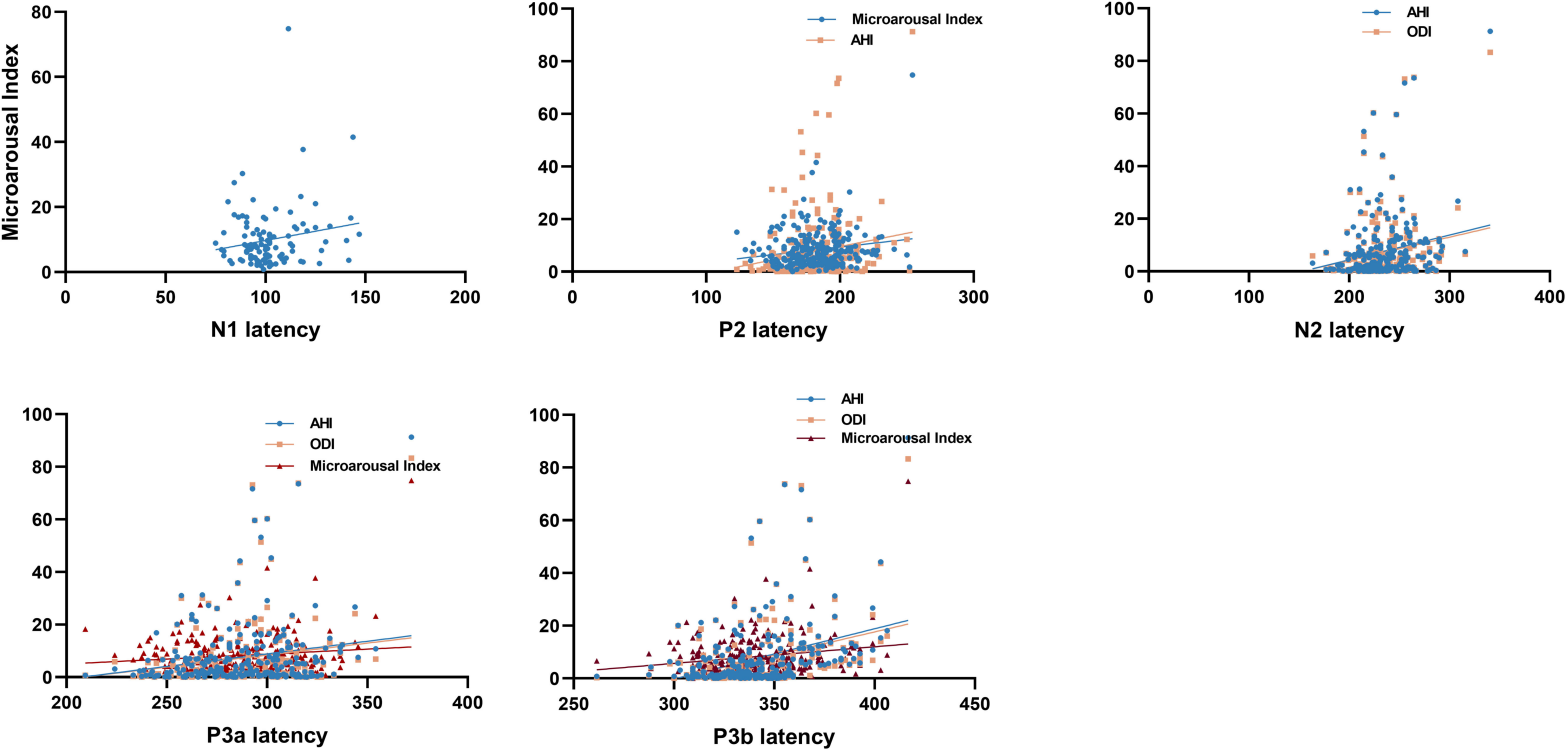
The correlation between P300 components and PSG metrics.

We found that a higher respiratory/arousal index was significantly associated with prolonged P300 latency in patients with MDD-OSA. This association was robust to adjustment for gender, age, BMI, education, smoking, alcohol consumption, and antidepressant use. Specifically, both AHI and ODI were independently associated with prolonged latencies of the N2, P3a, and P3b components. Additionally, a higher microarousal index was associated with increased latencies of the N1, P2, P3a and P3b components (all p < 0.05). Conversely, sleep architecture metrics (proportions of N1, N2, N3, REM, and wake stages) were not correlated with any P300 component latencies in the MDD-OSA group (all p > 0.05) (**Supplementary Table S2**, **Figure 5**).

## 4 Discussion

Major depressive disorder (MDD) and obstructive sleep apnea (OSA) are common comorbid conditions, and the presence of OSA may exacerbate cognitive impairment in MDD. This study aims to assess the impact of comorbid OSA on cognitive function in patients with MDD, using the P300 component as a neurophysiological marker. Additionally, it examines whether this impact is associated with impairments in sleep continuity-specifically, sleep architecture disruption and fragmentation-and nocturnal hypoxia. Our study observed that the MDD-MO and MDD-SO groups comprised older patients and were characterized by a higher percentage of males and BMI. The MDD-SO group was characterized by a shift toward lighter, more fragmented sleep compared to the MDD and MDD-MO groups, specifically manifesting as a higher N1 percentage, lower N3 and REM percentages, and an elevated microarousal index. Co-occurring OSA may exacerbate cognitive impairment in patients with MDD, primarily manifested as prolonged latencies of the N2, P3a, and P3b components. Furthermore, higher scores on the AHI, ODI, and the Micro-arousal index were each independently associated with more significant cognitive decline.

### 4.1 Clinical characteristics

In line with observations in the general OSA population (38, 39), our study identified older age and elevated BMI-but not gender or depression severity-as significant independent predictors of OSA comorbidity in this MDD cohort. The association with age may be attributed to a confluence of structural, functional, and regulatory changes (40). The link with BMI is particularly relevant in MDD, where factors such as chronic antidepressant use and lifestyle alterations increase obesity risk. A key consequence is the enlargement of peripharyngeal soft tissue, which predisposes the upper airway to collapse during sleep (41). Interestingly, male gender was not a significant risk factor in our cohort. A potential explanation is that most female participants were postmenopausal; the decline in protective hormones like estrogen and progesterone may mitigate the typical sex disparity in OSA prevalence (42).

### 4.2 Characteristics of sleep parameters

Research on sleep architecture changes in the OSA-depression interaction is limited but informative. An early study by Reynolds et al. (43) identified reduced REM sleep latency as a polysomnographic hallmark of depression, but noted that comorbid OSA could prolong it (43). In contrast, Bardwell et al. (44) focused on different metrics, reporting that depressed patients with OSA exhibited shorter sleep latency and a higher proportion of REM sleep compared to those without OSA (44). Our results reveal more severe sleep disturbances in patients with comorbid MDD and OSA compared to those with MDD alone. This is evidenced by: 1) disordered sleep architecture (reduced sleep efficiency, increased N1%, decreased N3% and REM sleep); 2) heightened sleep fragmentation (more frequent and prolonged awakenings), and 3) greater intermittent hypoxia (elevated ODI). Besides, to address the potential confounding effect of age, we performed a robustness check using age-matched subgroups. The overall findings remained consistent (**Supplementary Table S4**). Notably, however, the between-group differences in the proportion of N3 sleep and sleep efficiency became non-significant after age matching. This indicates that the observed reductions in N3 sleep and sleep efficiency among MDD patients with OSA are more likely attributable to age rather than to OSA per se. Therefore, the exacerbation of sleep disorders in these patients likely occurs through the following indirect mechanisms: 1) Hypoxia-Induced Arousal: Apnea-related oxygen desaturation stimulates carotid chemoreceptors, triggering brainstem arousal centers and consequent sleep fragmentation (45); 2) Autonomic-Endocrine Dysregulation: Recurrent obstruction leads to apnea, hypoxia, and HPA-axis activation. This propagates a state of physiological hyperarousal and elevated sympathetic tone, further fragmenting sleep (46); 3) Neuroinflammatory Pathways: Intermittent hypoxia induces a systemic inflammatory response. The resulting circulating inflammatory factors may then disrupt hypothalamic sleep-wake regulation, increasing sleep latency (47).

### 4.3 The presence of comorbid OSA alters P300 indexes in patients with MDD

As a neurophysiological index of cognitive processing, the P300 is of paramount importance for researching cognitive impairment in individuals with depression. The subcomponents of P300 index distinct cognitive processes: the N2 indexes attentional control and conflict monitoring (48, 49); the P3a marks the orienting response and initial signal evaluation (50); and the P3b underlies conscious target detection and working memory updating (51). This study found that, compared to the MDD group, patients with MDD-MO exhibited prolonged latencies of N2 and P3a, This suggests impaired attentional allocation and automatic processing of novel stimuli in the MDD-MO group. While a similar trend was observed in the MDD-SO subgroup, the difference was not statistically significant, likely due to its smaller sample size. Future studies with larger cohorts are needed to confirm this. In addition, we found that the P3b latency was significantly prolonged in both the MDD-MO and MDD-SO groups compared to the MDD group, this indicates that comorbid OSA impairs working memory updating and reduces information processing speed in patients with MDD. However, studies have demonstrated that the P300 component reflects distinct stages of information processing within a complex, interdependent cortical chain (52, 53). It should therefore be analyzed as an integrated entity, not in isolation (52, 53). In summary, comorbid OSA appears to disrupt the sequential stages of information processing in MDD, and the N2, P3a, and P3b components may not only reflect this disruption but also serve as potential biomarkers for the comorbidity, offering a window into its underlying neurocognitive mechanisms.

### 4.4 Hypoxia and fragmented sleep are key pathophysiological features that characterize the profile of further cognitive impairment in patients with MDD-OSA

The P300 component is influenced by multiple factors, such as age, which typically prolongs its latency (54). To address this, we employed a multivariate linear regression model, controlling for gender, age, body mass index, educational level, smoking status, alcohol consumption, and antidepressant use. Our findings demonstrated an association between hypoxemia/sleep fragmentation and delayed P300 latency in patients with MDD-OSA comorbidity. Specifically, in this comorbid group, higher oxygen desaturation index (hypoxemia) and microarousal index (sleep fragmentation) were significantly correlated with prolonged latencies of the P2, N2, P3a, and P3b latency. Notably, these correlations were absent in patients with MDD alone. Collectively, the findings position hypoxemia and sleep fragmentation as factors intimately associated with P300 abnormalities in the specific context of MDD-OSA comorbidity.

Hypoxia and sleep fragmentation are correlated with cognitive impairment in patients with co-occurring OSA and MDD, and this correlation may be attributed to their adverse impacts on brain structure and function. Ju et al. (58) reported reduced relative cerebral blood flow in the bilateral hippocampus and parahippocampal gyrus among elderly patients with OSA, which may stem from impaired cerebral autoregulation due to chronic apnea and severe hypoxemia (55). Key brain regions like the frontal cortex, parietal cortex, and hippocampus have high metabolic demands, making them particularly vulnerable to hypoxia (56, 57). Therefore, we propose that intermittent hypoxia may inflict central nervous system damage through three interconnected mechanisms: the spillover of peripheral inflammation into the brain, direct neuronal damage, and microglial activation. Specifically, peripheral inflammatory signals may reach the brain via the blood-brain barrier or vagal afferent pathways, subsequently inducing neuronal damage/apoptosis and stimulating microglial M1 polarization. Activated microglia then produce neurotoxic substances, including pro-inflammatory factors and reactive oxygen species (ROS) (58). Similar results have also been observed in animal models, hypoxia induces oxidative stress and inflammatory responses, which in turn result in heightened apoptosis and hippocampal atrophy (59).

In addition to intermittent hypoxia, sleep fragmentation activates inflammatory responses in both central and peripheral systems, This exacerbates neuroinflammation, contributing to neural damage. Meanwhile, sleep fragmentation disrupts metabolic homeostasis in the nervous system. It specifically elevates glutamatergic metabolic signals in cognitively critical regions, such as the raphe nucleus and hippocampal CA1 area, while concurrently impairing the white matter fiber integrity within the hippocampal CA1 (60). Based on the integration of our findings, we speculate that chronic intermittent hypoxia and sleep fragmentation represent pivotal, synergistic mechanisms underlying nocturnal CNS injury. Their convergence may specifically disrupt the functional integrity of frontal, hippocampal, and limbic regions-neural circuits critically involved in generating the P300 response (61–64). Therefore, The aforementioned pathological processes could collectively underlie the electrophysiological (prolonged P300 latency) and behavioral (cognitive decline) manifestations in MDD-OSA patients (65, 66). In contrast to the associations found with hypoxic and arousal indices, sleep architecture measures did not show statistically significant correlations with P300 latency in the current analysis. This indicates that disordered sleep architecture plays a more limited role in relation to the P300 in this comorbid population, whereas intermittent hypoxia and sleep fragmentation are the primary associated factors contributing to the observed electrophysiological alterations.

### 4.5 Limitations

There are several limitations in this study. First, Given the cross-sectional design of this study, the observed associations cannot be interpreted as causal relationships. Future longitudinal or intervention studies are needed to explore potential causal directions. Second, a key methodological limitation involves the simplified classification of behavioral variables, namely smoking and alcohol use. Although both were included as covariates (smoking status and binary alcohol consumption) to adjust for potential confounding, this approach fails to capture their multifaceted and dose-dependent influences. For smoking, residual confounding may persist because nicotine exerts acute stimulant effects while contributing to chronic vascular and neuronal injury. Similarly, the binary classification of alcohol use obscures potential dose-response relationships or the differential impacts of light versus heavy use on cognitive electrophysiology. Consequently, future studies should integrate detailed, quantitative assessments of these behaviors-such as intensity, duration, and consumption patterns-within a broader framework that also includes early-life adversity (e.g., ACE scores) and lifestyle factors. In addition, the sample size limited our statistical power to test for potential interactions between key variables. Future studies with larger samples are needed to investigate these potentially complex interrelated effects. Third, our polysomnographic data did not include transcutaneous or end-tidal carbon dioxide (CO_2_) monitoring, we cannot rule out the possibility that hypoventilation contributed to the observed sleep disturbances and cognitive deficits in this group. Future studies need to incorporate CO_2_ monitoring to clarify its role in the cognitive profile of depressed patients with MDD-OSA. Fourth, Due to the lack of a control group of patients with OSA who have no history of depression. Therefore, the association we observed between high OSA severity and cognitive decline cannot be regarded as a specific pathological mechanism of MDD. Future studies need to directly compare MDD patients with OSA, OSA patients without MDD, and healthy controls to dissect the disease-specific and general components of the OSA effect. Besides, While our design assessed patients in the acute phase and controlled for prior antidepressant use, detailed information on specific drug classes and dosages was not available. Future studies with prospective, pharmacotherapy-controlled designs are needed to dissect medication-specific effects. Fifth, the interpretation of cognitive function in this study is based solely on electrophysiological correlates-P300. While P300 latency is well-established indices of neural processing speed, they do not constitute a direct measure of behavioral cognitive performance in domains such as memory, executive function, or visuospatial skills. The associations observed here between sleep disturbances and P300 abnormalities. Therefore, future studies that incorporate comprehensive neuropsychological assessments alongside electrophysiological measures are warranted to establish a more complete picture of cognitive impairment in MDD-OSA comorbidity.

## 5 Conclusions

This study aimed to explore cognitive function via P300 in patients with MDD-OSA comorbidity and to elucidate the relationships between P300 latency and key nocturnal disturbances: sleep architecture disruption, intermittent hypoxia, and sleep fragmentation. These results indicate that the P300 component may offer a sensitive electrophysiological marker for cognitive dysfunction in MDD-OSA comorbidity. Furthermore, they suggest that intermittent hypoxia and sleep fragmentation are potential factors contributing to such damage.

## Data Availability

The raw data supporting the conclusions of this article will be made available by the authors, without undue reservation.
