# Supplementary material for: Cognitive impairment in comorbid MDD and OSA: the dual effects of intermittent hypoxia and sleep fragmentation
Source: Front Psychiatry. 2026 Jan 21;17:1687180. doi: 10.3389/fpsyt.2026.1687180 (PMC12868299; doi:10.3389/fpsyt.2026.1687180)
Supplement: Supplementary file 1 [file Supplementaryfile1.docx]

**Supplementary Information**

**Table S1.** The differences of PSG parameters among three groups

**Table S2.** Multiple linear regression analysis examining the association between polysomnography (PSG) metrics and ERP components

**Table S3.** Potential predictive factors for comorbid OSA in patients with MDD

**Table S4.** The disparities in polysomnographic parameters among the three groups subsequent to age matching

**Figure S1.** Participants flow chart.

**Table S1.** The differences of PSG parameters among three groups

| **Variables** | **MDD group (A)**  **（n=136）** | **MDD-MO group (B)**  **（n=75）** | **MDD-SO group (C)**  **（n=34）** | **P value** |
| --- | --- | --- | --- | --- |
| Sleep efficiency（%）,(mean±SD) | 88.25±6.93* | 85.39±8.62 | 84.45±8.62 | 0.011^a^ |
| ODI,M(QR) | 0.85(1.60)* | 6.80(5.10)*** | 22.35(19.38)** | <0.001^a^ |
| Proportion of N1 stage（%）,M(QR) | 11.23(7.25) | 11.22(9.29 )*** | 14.85(14.61)** | 0.001^a^ |
| Proportion of N2 stage（%）,M(QR) | 52.96(18.98) | 56.36(21.45) | 54.54(14.13) | 0.252 |
| Proportion of N3 stage（%）,M(QR) | 12.17(18.57) | 4.41(10.80)*** | 5.72(7.19)** | <0.001^a^ |
| Proportion of REM stage（%）,M(QR) | 12.66(9.59) | 10.88(8.92) | 9.13(11.26)** | 0.010^a^ |
| Proportion of waking period（%）,M(QR) | 6.31(6.08)* | 9.90(10.45) | 9.04(12.35) | 0.002^a^ |
| Microarousal Index,M(QR) | 5.95(6.00) | 6.40(7.50)*** | 10.60(9.68)** | <0.001^a^ |

Note:A: MDD group; B:MDD-MO group; C: MDD-SO group; a: Kruskal-Wallis H Test.

*A ≠ B,**A ≠ C,***B ≠ C.

ODI:Oxygen desaturation Index.

**Table S2.** Multiple linear regression analysis examining the association between polysomnography (PSG) metrics and ERP components

| **Dependent Variable** | **Independent Variable** | **B** | **SE** | **Beta** | **95% CI** |
| --- | --- | --- | --- | --- | --- |
| **N1(ms)** | AHI | 0.133 | 0.100 | 0.096 | (-0.064,0.330) |
|  | ODI | 0.116 | 0.102 | 0.082 | (-0.084,0.317) |
|  | Proportion of N1 stage(%) | -0.021 | 0.136 | -0.011 | (-0.288,0.246) |
|  | Proportion of N2 stage(%) | 0.098 | 0.083 | 0.080 | (-0.065,0.262) |
|  | Proportion of N3 stage(%) | -0.043 | 0.109 | -0.029 | (-0.258,0.171) |
|  | Proportion of REM stage(%) | -0.072 | 0.171 | -0.030 | (-0.409,0.266) |
|  | Proportion of waking period(%) | -0.139 | 0.153 | -0.062 | (-0.441,0.164) |
|  | Microarousal Index | 0.387 | 0.151 | 0.222 | (0.078,1.123)* |
| **P2(ms)** | AHI | 0.357 | 0.126 | 0.204 | (0.108, 0.605)* |
|  | ODI | 0.316 | 0.129 | 0.176 | (0.062, 0.569) |
|  | Proportion of N1 stage(%) | 0.196 | 0.173 | 0.078 | (-0.145, 0.536) |
|  | Proportion of N2 stage(%) | -0.065 | 0.106 | -0.042 | (-0.275, 0.145) |
|  | Proportion of N3 stage(%) | 0.100 | 0.139 | 0.053 | (-0.174, 0.374) |
|  | Proportion of REM stage(%) | -0.207 | 0.218 | -0.069 | (-0.637, 0.223) |
|  | Proportion of waking period(%) | -0.072 | 0.196 | -0.025 | (-0.459, 0.315) |
|  | Microarousal Index | 0.612 | 0.229 | 0.266 | (0.157, 1.067)* |
| **N2(ms)** | AHI | 0.376 | 0.150 | 0.181 | (0.081,0.670)* |
|  | ODI | 0.361 | 0.152 | 0.170 | (0.061, 0.661)* |
|  | Proportion of N1 stage(%) | 0.295 | 0.204 | 0.099 | (-0.107, 0.696) |
|  | Proportion of N2 stage(%) | -0.071 | 0.126 | -0.038 | (-0.319, 0.177) |
|  | Proportion of N3 stage(%) | 0.136 | 0.164 | 0.061 | (-0.188, 0.460) |
|  | Proportion of REM stage(%) | -0.269 | 0.256 | -0.077 | (-0.773, 0.235) |
|  | Proportion of waking period(%) | -0.256 | 0.232 | -0.076 | (-0.712, 0.200) |
|  | Microarousal Index | 0.500 | 0.294 | 0.173 | (-0.083, 1.084) |
| **P3a(ms)** | AHI | 0.417 | 0.147 | 0.205 | (0.128,0.707)* |
|  | ODI | 0.410 | 0.149 | 0.197 | (0.116, 0.705)* |
|  | Proportion of N1 stage(%) | 0.194 | 0.201 | 0.066 | (-0.203, 0.591) |
|  | Proportion of N2 stage(%) | -0.017 | 0.124 | -0.010 | (-0.262, 0.227) |
|  | Proportion of N3 stage(%) | 0.106 | 0.162 | 0.048 | (-0.214, 0.426) |
|  | Proportion of REM stage(%) | -0.039 | 0.251 | -0.011 | (-0.533, 0.456) |
|  | Proportion of waking period(%) | -0.434 | 0.227 | -0.132 | (-0.882, 0.014) |
|  | Microarousal Index | 0.678 | 0.258 | 0.259 | (0.166, 1.190)* |
| **P3b(ms)** | AHI | 0.593 | 0.128 | 0.315 | 0.342,0.845)** |
|  | ODI | 0.563 | 0.131 | 0.292 | (0.306,0.820)** |
|  | Proportion of N1 stage(%) | 0.344 | 0.179 | 0.127 | (-0.008, 0.697) |
|  | Proportion of N2 stage(%) | -0.077 | 0.111 | -0.046 | (-0.295, 0.141) |
|  | Proportion of N3 stage(%) | 0.058 | 0.145 | 0.028 | (-0.228, 0.343) |
|  | Proportion of REM stage(%) | -0.112 | 0.227 | -0.035 | (-0.560, 0.336) |
|  | Proportion of waking period(%) | -0.246 | 0.204 | -0.081 | (-0.648, 0.156) |
|  | Microarousal Index | 0.715 | 0.258 | 0.265 | (0.203, 1.227)* |

Note:Adjustment for age, gender, BMI, education level, smoking and drinking antidepressant use.

95% CI: 95% confidence interval;AHI:Apnea-hypopnea Index. RDI: Respiratory Distress Index.ODI:Oxygen desaturation Index.

*P＜0.05

**P＜0.01

**Table S3.** Potential predictive factors for comorbid OSA in patients with MDD

| **Variable** | **β** | **SE** | ***P*** | **OR** | **95%CI** |
| --- | --- | --- | --- | --- | --- |
| Age | 0.070 | 0.013 | 0.000 | 1.073 | 1.046,1.101 |
| BMI | 0.243 | 0.051 | 0.000 | 1.274 | 1.153,1.408 |
| Gender,Male | 0.445 | 0.335 | 0.184 | 1.560 | 0.809,3.009 |
| HAMD | -0.015 | 0.017 | 0.387 | 0.986 | 0.953,1.019 |

**Table S4.** The disparities in polysomnographic parameters among the three groups subsequent to age matching

| **Variables** | **MDD group (A)**  **（n=34）** | **MDD-MO group (B)**  **（n=75）** | **MDD-SO group (C)**  **（n=34）** | **P value** |
| --- | --- | --- | --- | --- |
| Sleep efficiency（%）,(mean±SD) | 81.32±8.69 | 85.39±8.62 | 84.45±8.62 | 0.088 |
| ODI,M(QR) | 1.55(1.98)* | 6.80(5.10)*** | 22.35(19.38)** | <0.001a |
| Proportion of N1 stage（%）,M(QR) | 12.58(6.27) | 11.22(9.29 )*** | 14.85(14.61)** | 0.010a |
| Proportion of N2 stage（%）,M(QR) | 53.06(18.87) | 56.36(21.45) | 54.54(14.13) | 0.717 |
| Proportion of N3 stage（%）,M(QR) | 5.63(11.33) | 4.41(10.80) | 5.72(7.19) | 0.789 |
| Proportion of REM stage（%）,M(QR) | 9.82(6.55) | 10.88(8.92) | 9.13(11.26) | 0.491 |
| Proportion of waking period（%）,M(QR) | 15.85(11.71) | 9.90(10.45) | 9.04(12.35) | 0.076 |
| Microarousal Index,M(QR) | 6.30(7.50) | 6.40(7.50)*** | 10.60(9.68)** | 0.003a |

Note:Through 1:1 propensity score matching on age

A: MDD group; B:MDD-MO group; C: MDD-SO group; a: Kruskal-Wallis H Test.

*A ≠ B,**A ≠ C,***B ≠ C.

ODI:Oxygen desaturation Index.

**
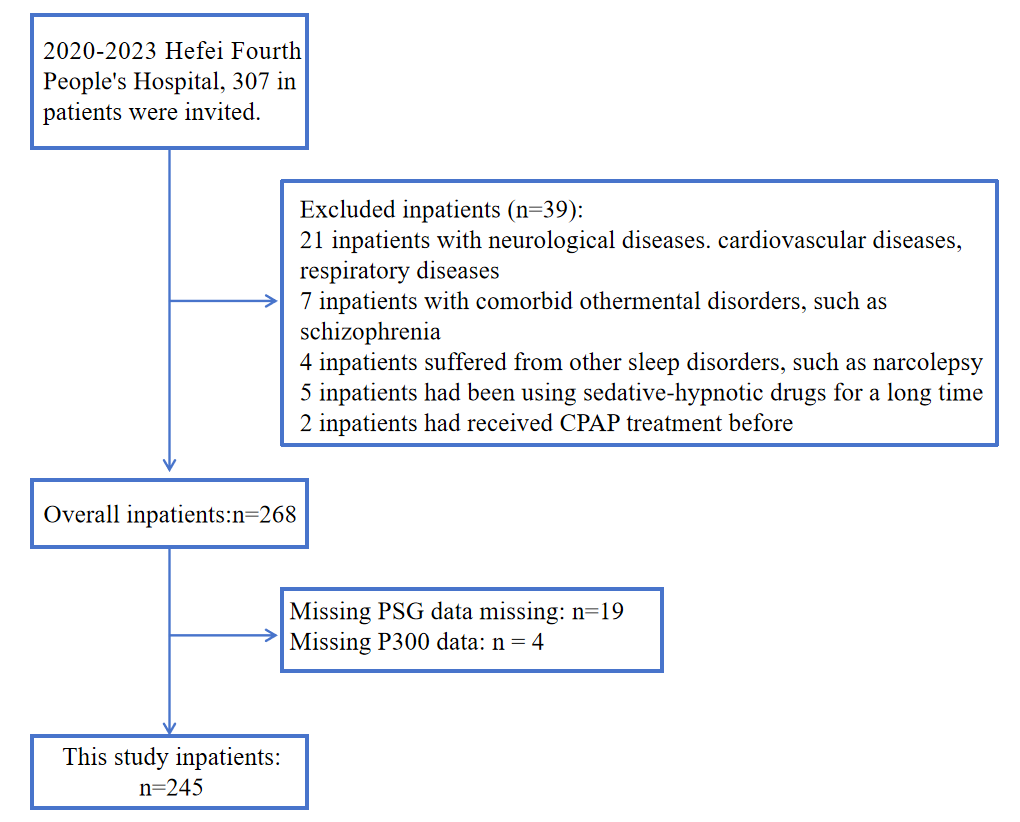
**

**Figure S1.** Participants flow chart.
